# Right atrial appendage firing in atrial fibrillation

**DOI:** 10.3389/fcvm.2022.997998

**Published:** 2022-10-17

**Authors:** Florian Baptiste, Jérôme Kalifa, Cyril Durand, Edouard Gitenay, Michel Bremondy, Anis Ayari, Nicolas Maillot, Antonio Taormina, Aicha Fofana, Guillaume Penaranda, Sabrina Siame, Clément Bars, Julien Seitz

**Affiliations:** ^1^Hôpital Saint Joseph, Marseille, France; ^2^Department of Cardiology, Brown University, Providence, RI, United States; ^3^ADRIS_Médipôle Lyon-Villeurbanne, Lyon, France; ^4^Département Statistique, Laboratoire Alphabio, Marseille, France

**Keywords:** AF driver, dispersion, right atrial appendage, AF ablation, tailored ablation

## Abstract

**Background:**

The role of atrial fibrillation (AF) drivers located at the left atrium, superior vena cava, crista terminalis and coronary sinus (CS) is well established. While these regions are classically targeted during catheter ablation, the role of right atrial appendage (RAA) drivers has been incompletely investigated.

**Objective:**

To determine the prevalence and electrophysiological characteristics of AF driver’s arising from the RAA.

**Materials and methods:**

We conducted a retrospective analysis of clinical and procedural data of 317 consecutive patients who underwent an AF ablation procedure after bi-atrial mapping (multipolar catheter). We selected patients who presented with a per-procedural RAA firing (RAAF). RAAF was defined as the recording of a sustained RAA EGM with a cycle length shorter than 120 ms or 120 < RAAF CL ≤ 130 ms and ratio RAA CL/CS CL ≤ 0.75.

**Results:**

Right atrial/atrium appendage firing was found in 22 patients. The prevalence was estimated at 7% (95% CI, 4–10). These patients were mostly men (72%), median age: 66 yo ± 8 without structural heart disease (77%). RAAFs were predominantly found in paroxysmal AF patients (63%, 32%, and 5% for paroxysmal, short standing and long-standing AF, respectively, *p* > 0.05). RAAF median cycle length was 117 ms ± 7 while CS cycle length was 180 ms ± 10 (*p* < 0.01).

**Conclusion:**

In 317 consecutive AF ablation patients (22 patients, 7%) the presence of a high-voltage short-cycle-length right atrial appendage driver (RAAF) may conclusively be associated with AF termination. This case series exemplifies the not-so-uncommon role of the RAA in the perpetuation of AF.

## Introduction

The role of the left atrium (LA) in the pathogenesis of atrial fibrillation (AF) is well established by electrophysiological (1–4) and histological studies (5–7). Classically, AF catheter ablation mostly targets left atrial regions (8–10).

Still the right atrium (RA) is known to play a crucial role in AF pathogenesis: Zarse et al. showed in a rabbit model that sustained AF may be observed in the presence of RA dilation without LA dilation (11). In addition, patient-tailored, electrogram-based ablation approaches have highlighted the role of right-sided structures such as the superior vena cava, crista terminalis, septum or coronary sinus (CS) ostium (2, 12–14) for the perpetuation of AF. Marcus and al exposed a case of a RAA « rotor » identified by non-invasive and invasive mapping of a paroxysmal AF after 2 failed ablation procedures. Targeted ablation of this EGM also led to AF termination and the absence of recurrence after an 18 months follow up (15).

Classical electrogram-based ablation workflows require LA mapping/ablation completed with RA mapping/ablation (16). This workflow allows for the observation of rapid AF drivers exquisitely located within the right atrial appendage (RAA). Here, we present a consecutive patient series, in which the epidemiological, electrophysiological and ablation characteristics of RAA AF drivers (RAAFs) have been examined.

## Materials and methods

### Patients

We conducted a retrospective analysis of clinical and procedural data of patients (2015–2019) who underwent an EGM-based AF ablation procedure in two centers: Hôpital Saint Joseph, Marseille, France, Medipôle Lyon-Villeurbanne, Lyon, France.

These corresponded to single-physician patient series at both centers. Consecutive patients who underwent a patient-tailored ablation of AF guided by spatio-temporal dispersion of electrograms (16) were included in the analysis. Paroxysmal, persistent, long standing persistent AF patients undergoing either an index or a redo procedure were considered. Clinical, electrophysiological features and procedural data were extracted for all patients. Patients who presented with a per-procedural RAA firing (RAAF) pattern were compared with the ones without RAAF (no-RAAF patients). Patients having undergone an anatomical-based catheter ablation—i.e., without bi-atrial mapping — or patients with incomplete medical and procedural data were excluded. The study was approved by the institutional ethics committees and all patients provided signed informed consent.

### Procedure

As described previously (16), a decapolar reference catheter was positioned within the coronary sinus (CS) and a baseline mapping in LA or both atria was performed during AF with a multi-electrode catheter (PentaRay^®^, Biosense Webster, Inquiry Optima^®^ or HD-Grid^®^ Abbott). The multi-electrode catheter was sequentially positioned in various regions of the LA and RA. At each location, the catheter was maintained in a stable position. The operator visually analyzed EGMs directly on the EP recording system [PruckaCardioLab^®^ recording system (GE Healthcare, Wauwatosa, WI)] and looked for dispersion areas i.e., electrograms exhibiting spatio temporal dispersion as reported previously (16). In regions where dispersion was found and/or when the catheter was not in a stable position, acquisitions were repeated multiple times. For patients in sinus rhythm (SR) at the outset of the procedure, AF was induced by rapid atrial pacing using the CS catheter (500–180 ms). If AF was not inducible, isoproterenol (bolus from 0.02 to 0.04 mg in order to obtain a sinus rate > 100 bpm) was infused.

Once the initial atrial mapping was obtained, the choice of the first dispersion region to ablate was left at the operator’s discretion. The procedural endpoint was AF termination/sinus rhythm conversion. If AF did not terminate after ablation of the left or bi-atrial dispersion area regions, a re-mapping or a cardioversion was performed at the physician’s discretion. If AF was induced at the beginning of the procedures, AF non-inducibility was tested after sinus rhythm conversion. Inducibility protocol was the following: AF reducibility was tested by rapid atrial pacing (500–180 ms) in the proximal and distal CS with the addition of isoproterenol to induce AF when necessary.

**FIGURE 1:**
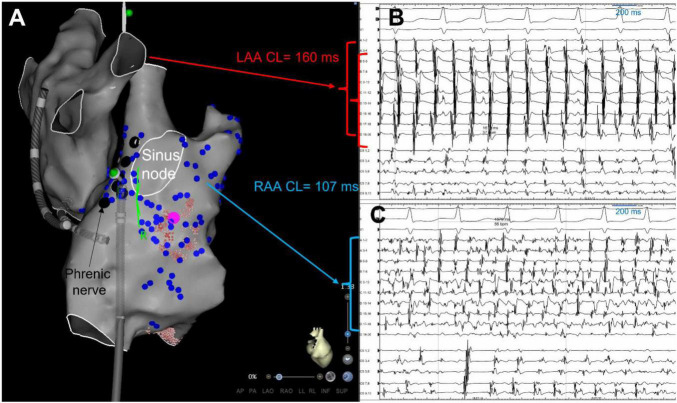
Right atrial appendage firing (RAAF) ablation limited by phrenic nerve and sinus node locations. Biatrial 3D shells in the right lateral view (panel **A**), in a patient with paroxysmal AF recurrences despite a previous PVI. AF was induced by atrial pacing, PVs were not connected, and biatrial mapping highlighted a rapid driver in the right atria, located at the appendage/lateral wall area (blue dots = dispersion, cycle length = 107 ms – panel **B**), while the left atrium was bystander (no dispersion, cycle length = 160 ms – panel **C**). Ablation of this driver was limited by the location of the sinus node (white circle) mapped in sinus rhythm and the phrenic nerve (black dots) located by pacing. Sinus rhythm was restored by ablation at this area (pink dot) and AF was not inducible anymore. CL, cycle length, LAA, left atrial appendage, RAA, right atrial appendage.

Radiofrequency energy was applied (25–50 W) with irrigated ablation catheter (ThermoCoolSmartTouch^®^ Biosense Webster or TactiCath^®^ Quartz Abbott) at any atrial location including the PV antrum and the CS, while no ablation was performed inside the PVs. A contact force of 5 grams was considered a minimum to deliver RF energy at any location. Point by point applications were performed (no dragging). If two ablated areas were very close (<1 cm) they were connected by RF applications. Additional pulmonary vein (PV) isolation or linear ablation were performed at the physician’s discretion. No ablation of non PV triggers was performed, only substrate based ablation as described previously (16). For RF applications within the RAA, power settings were 40–50 watts with ablation index/LSI target at 500/5.5. Pacing maneuvers were used to locate the phrenic nerve and we avoided applications in the sinus node area, which was mapped in sinus rhythm when feasible (Figure 1).

### Study endpoints

The primary endpoints of this study were to describe the epidemiological, electrophysiological, and procedural features of patients presenting a RAAF.

The secondary endpoint was the identification of predictive factors of the RAAF.

### Right atrial appendage firing definition

Right atrial appendage firing is defined as a short-cycle-length, relatively high-voltage (higher voltage than the coronary sinus EGM voltage at the same time and minimal value of 0.2 mV), sustained multipolar EGM pattern recorded within the RAA and/or in its close vicinity. More specifically, RAAF are characterized as follows:

- Duration > 1 min.
- RAA cycle length (CL) < 120 ms or 120 < RAAF CL ≤ 130 ms and a ratio RAA CL/CS CL ≤ 0.75.

### Electrogram analysis

Cycle length (ms) and voltage (mV) were measured as the average of ten consecutive EGMs on a General Electric Prucka Cardiolab recording system.

### Follow up

Conventional follow-up visits and 24-h Holter monitors were scheduled at 3, 6, 12, 18 months, then every year of follow-up and in case of symptoms at the outpatient clinic or with the external referent cardiologist. All patients were instructed to contact the study center if they experienced palpitations or another symptom suggesting of arrythmia recurrence. Data were retrospectively extracted from the medical file, also by contacting external cardiologists and the patients.

### Statistical analysis

Quantitative data were reported using medians and interquartile ranges (IQR). Qualitative data were reported using frequencies and percentages. Non-parametric Wilcoxon test was used to compare medians among groups, and chi-square test was used to compare frequencies. *P*-values were considered significant at an alpha level of 0.05. All calculations were performed using SAS V9.4 software (SAS Institute Inc., Cary, NC).

## Results

### Population characteristics and right atrial appendage firing prevalence

Between June 2015 and February 2020, 467 single-physician consecutive AF ablations were considered for analysis. Ninety-seven patients were excluded due to a lack of available data or a non-biatrial mapping, while 53 were excluded because an anatomical ablation was performed in sinus rhythm. Thus, 317 consecutive procedures with systematic biatrial AF mapping were included. Overall, 22 patients with RAAF were identified. The prevalence of RAAF was estimated at 7% (95% CI, 4–10). 9/22 RAAF patients (41%) were redo ablation procedures. The baseline characteristics of RAAF patients compared to non-RAAF patients are summarized in Table 1.

**TABLE 1:** Right atrial appendage firing (RAAF) and non-RAAF patients baseline characteristics.

Variables	RAAF patients (*n* = 22)	Non-RAAF (*n* = 295)	*p*
**Clinical features**			
Age, median [IQR], years	66 [55–71**]**	67 [60–73**]**	0.213
Sex, male n (%)	16 (72%)	230 (78%)	0.120
Paroxysmal AF n (%)	14 (63%)	89 (30%)	0.154
Short standing persistent AF n (%)	7 (32%)	150 (51%)	0.154
Long standing persistent AF n (%)	1 (5%)	56 (19%)	0.154
AF history, median [IQR], years	5 [1–10**]**	12 [3–13**]**	0.483
**Previous AF ablation, n (%)** **0** **1** **2** **3** **4**	13 (59%) 4 (18%) 3 (14%) 1 (5%) 1 (5%)	233 (59%) 47 (16%) 12 (14%) 2 (<1%) 1 (<1%)	0.355
Hypertension, n (%)	11 (50%)	156 (53%)	0.770
Diabetus mellitus, n (%)	1 (5%)	38 (13%)	0.334
Obstructive sleep apnea, n (%)	4 (18%)	74 (25%)	0.567
Coronary artery disease, n (%)	3 (14%)	24 (8%)	0.412
Structural heart disease, n (%)	5 (23%)	103 (35%)	0.353
LVEF, median (IQR),%	60 [55–60)	60 (55–60)	0.455
LA volume, median [IQR], mL	152 [120–170**]**	155 [131–184**]**	0.233
RA volume, median [IQR], mL	137 [120–173**]**	136 [107–165**]**	0.597
**Procedural data**			
Procedure time, median [IQR], (min)	178 [136–196**]**	165 [132–200**]**	0.554
Xray exposure, median [IQR], cGy/m^2^	2430 [1083–3433**]**	1426 [635–3722**]**	0.305
AF induction, n (%)*	19 (86%)	153 (52%)	**0.0010**
Isoproterenol induction, n (%)*	13 (65%)	86 (29%)	**0.0034**
RF time, median [IQR], min	54 [38–88)	46 [32–59**]**	**0.0279**

LA, left atrium; RA, right atrium, RAAF, right atrial appendage firing; AF, atrial fibrillation; LVEF, left ventricular ejection fraction; RF, radiofrequency; *: data available for 20 patients in the RAAF group. Bold values are for *p* < 0.05 stastitical significance.

### Ablation procedure and follow up

**FIGURE 2:**
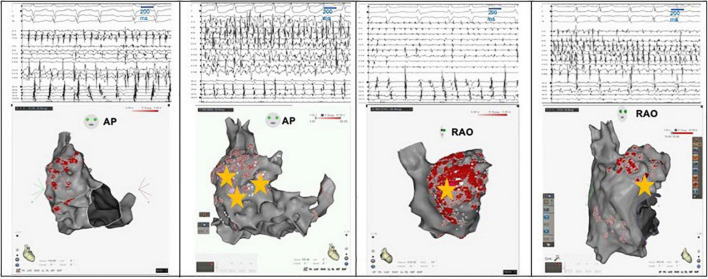
Examples of RAAF in four different patients. A multi-spline catheter is positioned within the right atrial appendage and a decapolar catheter within the coronary sinus. Electrogram activations recorded within the right appendage are *notably fast* (cycle length around 100 ms significantly shorter than the one measured in coronary sinus) and *fractionated*. Three dimensional shells of the right atrium are presented either in the antero-posterior (AP) view or right oblique anterior (RAO) view. Ablation areas are presented with visitags “grids” (pink/red areas) and yellow stars represent termination sites.

In RAAF patients, AF was more often induced using an isoproterenol bolus than in non-RAAF patients (Table 1). RAAF EGM were mapped and ablated either directly within and around the appendage. Because of an intricate anatomy in the area, ablation was at times rendered challenging by high voltage, catheter instability, the need for high power applications (between 40 and 50 W) and relative extensive ablation. A phrenic nerve mapping by pacing maneuvers was systematically performed, and ablation applications close to the sinus node location were avoided (Figure 1). In some patients, the avoidance of the phrenic nerve or sinus node had operators also ablate at the crista terminalis or right atrial free wall area (Figures 1, 2). The total procedure duration was similar between RAAF and non-RAAF patients; 178 [136–196] min vs. 165 [132–200] min, *p* = 0.55. The total radiofrequency time was significantly higher in RAAF patients than in non-RAAF patients; 54 [38–88] vs. 46 [32–59] min, *p* = 0.0279. In RAAF patients, the average time between the first radiofrequency application in the RAAF area and the end of procedure was 39 ± 28 min.

In one patient, a cryoballoon ablation catheter was needed to completely shut down the RAAF (after 2 failed procedures using catheter ablation and one 8 mm-tip RF catheter).

Ablation within the RAA led to AF termination in 21/22 patients (93%): AF conversion directly to sinus rhythm in *n* = 11 or atrial tachycardia in *n* = 4. In the non-terminating patient, a transient organization into atrial tachycardia was observed after electrical isolation of the RAA. AF, however, rapidly recurred and converted to sinus rhythm after ablation of left atrial appendage driver.

In one patient, ablation at the RAAF area led to an asymptomatic transient sinus node dysfunction which did not require pacemaker implantation. One cardiac hemopericardium occurring several days after the procedure was observed and was successfully evacuated percutaneously. In this case RF applications had been performed at the PV regions, the LA anterior wall, the RAA/crista terminalis area (leading to AF regularization into stable AT), the LA roof (for roof-dependent flutter), the lateral mitral isthmus and the distal CS (for peri-mitral flutter), the cavo-tricuspid isthmus (for common flutter) and the left and right septum (for localized AT). In terms of hemopericardium, a trend toward but no statistical difference was observed in comparison to non-RAAF patients (1/22 vs. 0/295, *p* = 0.07) with the limitation of unbalanced groups. No phrenic nerve palsy, persistent sinus node dysfunction or other major complications were observed.

A follow up was available for 19/22 patients (3 lost to follow up) at 24.5 ± 16 months. Freedom from AF after one procedure was achieved for 69% (12/19) of the patients. In two of the remaining patients, recurrence of AF was directly imputable to a recurrence of RAAF as RAAF recurrence was observed during an additional procedure and its ablation led to AF termination. At the end of the follow up 74% (14/19) were in stable sinus rhythm after 1.7 ablation procedures/patient.

### EGM analysis

**FIGURE 3:**
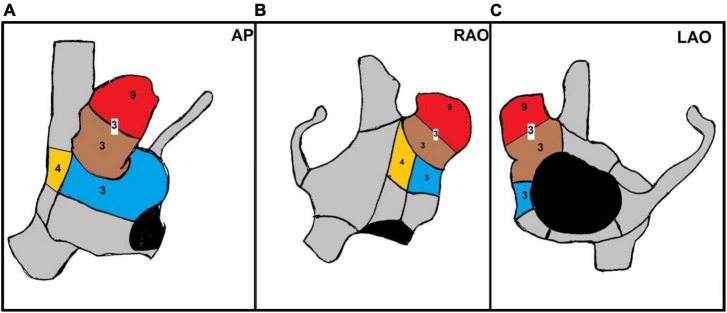
Anatomical distribution of AF termination sites in RAAF patients. Three dimensional shells of the right atrium [panel **A:** antero posterior view (AP), panel **B:** right anterior oblique (RAO) 30° view, panel **C:** left anterior oblique (LAO) 30° view]. Colored sections show anatomical regions of AF termination sites during ablation with the corresponding patients number. In red the RAA apex, in brown the RAA base, in blue the upper half of the right atrial free wall, in yellow the upper half of the crista terminalis. In three patients several AF termination sites were observed both in the apex and base of the RAA. In one patient, transient organization into atrial tachycardia was observed after RAA electrical isolation during ablation at the base.

**FIGURE 4:**
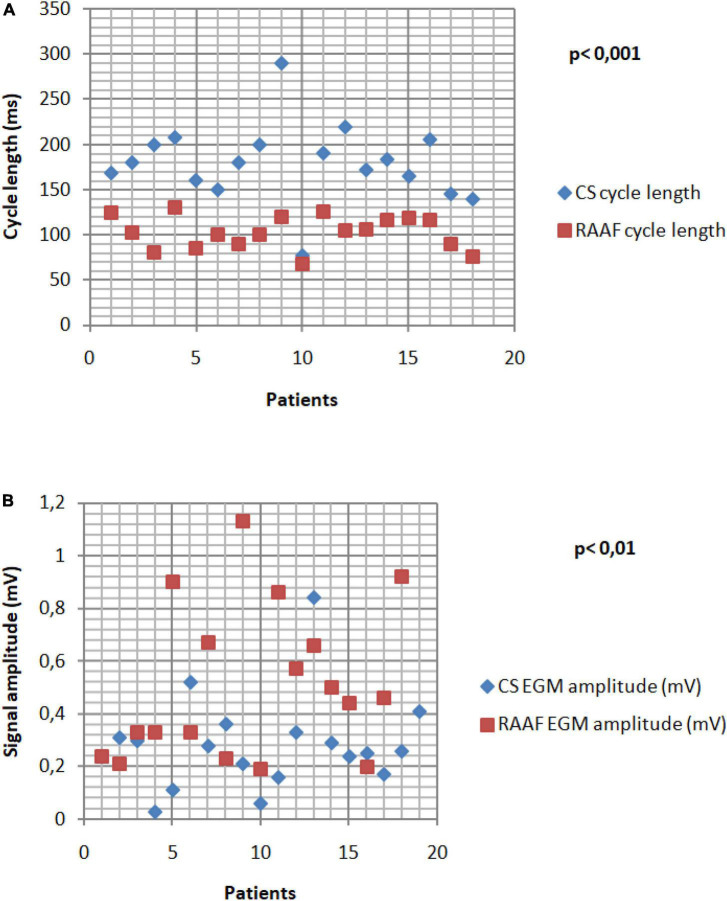
Right Atrial Appendage Firing (RAAF) EGM analysis. The cycle length analysis shows fast drivers with a mean cycle 1.94 shorter than the CS CL (*p* < 0.001) **(A)**. The RAAF cycle lengths were found between 76 and 130 ms. High voltage signals observed in the thick areas of the RAA and crista terminalis **(B)**. All the data were calculated as the average of ten consecutive measures.

In all patients, these EGM were located within the RAA and in its close vicinity: in 15 patients EGM were observed in the RAA only (9 at the RAA base, 3 at the RAA apex and 3 at both), in 7 patients the upper half of the crista terminalis (4 patients) and the right atrial free wall (3 patients) were also involved (Figures 2, 3). The multipolar catheter EGM analysis showed dispersion pattern characterized by either non-fractionated rapid fire or almost continuous poorly- fractionated signal (Figures 3, 4). The pattern showed temporal stability in all patients (the same EGM was recorded at 10 min intervals or more in all procedures).

Right atrial appendage firing was characterized by a very short cycle length, significantly shorter than the CS CL (117 ± 7 ms vs. 180 ± 10 ms *p* < 0.001, Figure 1A) and showed higher voltage in comparison to CS voltage [0.45 ± 0.21 mV vs. 0.27 ± 0.8 mV, *p* < 0.01)] (Figure 4).

### Predictive factors of right atrial appendage firing

**FIGURE 5:**
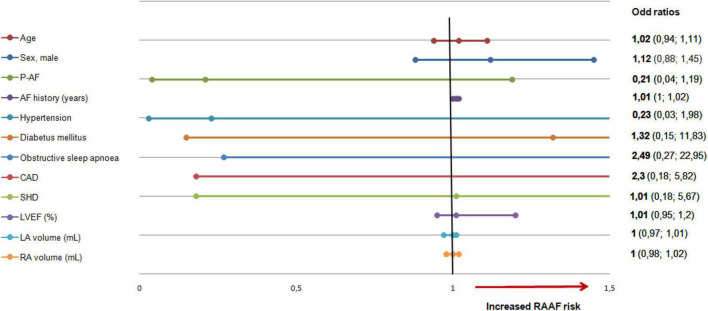
Right atrial appendage firing predictive factors. Clinical factors tested for RAAF. None of the studied factors were significantly associated with RAAF. LA, left atrium; RA, right atrium, RAAF, right atrial appendage firing; AF, atrial fibrillation, CAD, coronary artery disease; SHD, structural heart disease; LVEF, left ventricular ejection fraction.

None of the relevant clinical features studied were found predictive of RAAF (Figure 5 and Table 1).

## Discussion

### Main findings

While AF has been known to be maintained by left-sided or right-sided drivers (1–6, 12–14) the role of the right atrial appendage in the initiation and maintenance of AF has been incompletely investigated. Here, we report that a substantial group of patients undergoing AF ablation presents with critical RAA firing. This represented overall 22 patients out of 317 (prevalence = 7%). RAAFs were identified as sustained multipolar EGMs exhibiting high voltage/short CL. On average, patients with RAAFs required longer RF application but ablation was overall effective in terms of per-procedural AF termination and long-term outcomes.

### Anatomical structures harboring atrial fibrillation triggers/drivers

The description of anatomical structures specifically involved in the initiation and perpetuation of AF has been extensively investigated (1, 17–19). Left and right sided atrial structures such as ligament of Marshall (20), left atrial appendage (21), coronary sinus, Eustachian ridge or the crista terminalis (22) have shown potential arrhythmogenic activity. Either a tailored (16, 18) or the systematic (23, 24) targeting of such structures during ablation procedures, have shown results in preventing AF or atrial tachycardia (AT) recurrences.

### Right atrial appendage firings as potential ablation targets

The presence of RAAFs represents a potentially novel target for ablation. RAAFs are easily recognizable as they are characterized by high voltage rapid firing and/or continuous fractionated signal in all patients. Importantly, the regional extent of RAAFs constantly includes the RAA, while it inconstantly extends to the crista terminalis and right atrial free wall. In most patients exhibiting a RAAF, their AF-driving ability may be confirmed as in this series the left atrium was activated at a robustly longer cycle length and that the ablation of RAAFs led to AF termination— except in one patient. Unexpectedly, RAAFs were mostly found in paroxysmal AF patients after isoproterenol infusion (65%) thus highlighting a potentially preponderant role of the RAA and AF episodes relapse after PVI.

Here, RAAFs were found in patients ablated by three operators from two centers, giving credence to the reproducibility of the observation. Overall, the prevalence of RAAF was 7% (95% CI, 4–10) and 25% of RAAF were observed despite previous complete pulmonary vein isolation. By comparison, previous reports have indicated that RAA triggers were found in 0.33% of the procedures but RAA is a known source of 2–3% of AT (22). Mechanistically, we recognize that the right atrial ganglionated plexi, known to be implicated in AF pathogenesis (25–28) or RAA fibrosis might have underlied the RAAF activity (29).

### A new area of interest for ablationists – Specific technical challenges

The role of the RAA in the maintenance of AF is not well-known to ablationists. Refractory AF patients especially when PVI failed might benefit from an exhaustive bi-atrial mapping, with a cautious analysis of RAA signals. In the present case series, the targeting of the RAAF was technically challenging due to nearby anatomical structures (phrenic nerve, sinus node) and to the particularly thick and trabecular anatomy of the RAA. As a result, total RF time was significantly higher in patients presenting RAAF compared to non-RAAF patients despite high power RF applications (40–55 W). Despite these technical challenges, ablation of RAAF led to AF termination in all patients except for one. Also, ablation of RAAF led to a 1 year freedom from AF rate of 69% without additional procedure and 74% of stable SR after 1.7 procedure/patient. Half of AF recurrences were directly linked to a RAAF recurrence. These data suggest the crucial role of RAAF in AF perpetuation. Still, the exact impact and safety of RAAF ablation on long term clinical outcomes will need to be investigated in larger cohorts. Finally, new ablative energies such as pulsed field current (30) may represent promising options for RAAF ablation.

### Study limitations

None of the studied clinical features were predictive of RAAF. On the one hand, this may have resulted from a lack of statistical power. On the other, this underlies the need for a systematic bi-atrial mapping so that all critical structures and drivers involved in AF perpetuation are uncovered.

Also, the retrospective aspect of our work led to the exclusion of 97 patients due to lack of data or non-systematic biatrial mapping introducing a potential selection bias. The follow up data was recorded retrospectively. This study was not designed to evaluate long term outcomes of ablation in RAAF patients. Patients were enrolled in two expert centers by three physicians, questioning whether our findings are representative of real-life populations. In patients with RAAF, ablationists started the ablation in the LA and completed their set with ablation of RAAF. Future investigations will be needed to determine the impact of ablating RAAF first. Finally, investigation of the presence of additional extra-PV triggers with provocative maneuvers (19) was not performed. Such strategy would have provided an opportunity to examine whether RAAF regions overalp with other non-PV triggers. This will need to be investigated in future works.

## Conclusion

We identified and described a new AF driver with a very specific pattern located within the RAA in 22 patients. The driver was found mainly in paroxysmal AF even when PVI were isolated and though challenging its ablation seemed to be safe and effective for AF termination. This work suggests that bi-atrial mapping should be performed in case of paroxysmal and persistent AF recurrence despite a persistent PV isolation in order to identify a potential RAAF. More data are needed to identify predictors, long term safety and efficacy outcomes of RAAF ablation.

## Data availability statement

The original contributions presented in this study are included in the article/supplementary material, further inquiries can be directed to the corresponding author.

## Ethics statement

The studies involving human participants were reviewed and approved by the Hôpital Saint Joseph. The patients/participants provided their written informed consent to participate in this study.

## Author contributions

FB and JS contributed to the conception, design of the study, and writing. FB wrote the first draft of the manuscript. GP performed the statistical analysis. JK contributed to the writing. SS contributed to the submission. All authors contributed to the data collection and manuscript revision, read, and approved the submitted version.

## Abbreviations

AAD: antiarrhythmic drug
AF: atrial fibrillation
AFCL: atrial fibrillation cycle length
AT: atrial tachycardia
CFAE: complex fractionated atrial electrogram
CL: cycle length
CS: coronary sinus
LA: left atrial/atrium
PV: pulmonary vein
PVI: pulmonary vein isolation
RA: right atrial/atrium
RAA: right atrial/atrium appendage
RAAF: right atrial/atrium appendage firing
RF: radiofrequency
SR: sinus rhythm.
